# Hearing loss in people with HIV/AIDS and associated factors: an integrative review

**DOI:** 10.5935/1808-8694.20130042

**Published:** 2015-11-02

**Authors:** Luciana Ferreira Cardoso Assuiti, Gabriela Marcellino de Melo Lanzoni, Fabiana Cristine dos Santos, Alacoque Lorenzini Erdmann, Betina Hörner Schlindwein Meirelles

**Affiliations:** a MSc in Medical Sciences. Speech and Hearing Therapist; b PhD student in Nursing - UFSC; c Nursing Student - UFSC. CAPES scholarship holder; d PhD in Nursing Philosophy - Graduate Program - UFSC. Full Professor of the graduate program - UFSC; e PhD in Nursing Philosophy - Graduate Program - UFSC. Adjunct Professor of the undergraduate and graduate programs - UFSC. Federal University of Santa Catarina - UFSC

**Keywords:** audiology, deafness, hearing loss, HIV, nursing

## Abstract

**Abstract:**

The current scientific literature reports on the incidence of hearing impairments due to HIV/AIDS, and the hearing changes can occur due to damage to the outer, middle or inner ear. Thus, it is important to study how these changes occur, the hearing loss and their associations with the HIV/AIDS infection.

**Objective:**

To identify the factors related to hearing loss in people with HIV/AIDS in the global scientific literature.

**Method:**

Study carried out an Integrative Review of the Literature. The key words used were: hearing loss, hearing disorders and deafness, separately associated to the keyword HIV on PUBMED, ScIELO, LILACS and ISI databases. We used complete original papers, of free access, in English, Spanish, French and Portuguese. Thirteen quantitative studies from 1994-2010 were selected.

**Conclusion:**

We did not find any strong direct association between anti-retroviral therapy and hearing loss; however, there are indications of hearing loss in the population studied, and their associations and causes need to be better investigated.

## INTRODUCTION

In recent years, there has been promising developments in world initiatives to approach the AIDS epidemics, including a greater access to efficient treatment and prevention programs^1^. Notwithstanding, the AIDS issue remains as one of the most important health care challenges for world public health.

Considering such challenges, treatment with antiretroviral (ARV) medication is one of our greatest allies in controlling disease progression. These drugs started to be used worldwide by means of a mono-therapy with Zidovudine, in 1987, until we reached a point of having a combination of drugs, known as TARV^2^, ^3^. In Brazil, TARV started in 1991, and five years later a law was approved, making it mandatory the free distribution of the drug^4^.

Treatment with ARV medication has been the most important factor to control AIDS evolution, its chronicity and to guarantee the survival of those infected.

Considering HIV's specificity, which affects the immune system, favoring the incidence of diseases which prey on low immunity^5^, the TARV used to prevent these diseases causes intense or undesirable side effects, which is a problem for treatment. Although the progress in drug therapy has brought about a reduction in mortality and morbidity, there are a number of repercussions arising from it, such as the array of adverse effects associated with the ARVs, which causes a negative impact on the quality of life of those individuals who depend on the treatment^6^. Thus the need for healthcare professionals to pay the proper attention to these individuals, providing information about the potential side effects of each medication prescribed and the implementation of maneuvers in order to reduce the incidence of undesirable side effects. We also stress the importance of instructions concerning the concurrent use of other medications which may increase undesirable side effects^7^.

Studies have proven that some ARV medications may have potential ototoxic effects and cause hearing loss, and they point to a possible association with hearing loss in the central hearing system^7^ caused by the direct action of the virus, which in many cases is shown by otoneurological signs and symptoms presented by or reported by the patients, such as hearing loss, tinnitus and dizziness^8^, ^9^.

The hearing loss may be described as any reduction in hearing and/or in the person's capacity to detect speech or ambient sounds, regardless of cause, type of degree. This may happen at different moments in life, during gestation or delivery, in childhood, adult life or old age^10^. It may also be classified by type (conductive, sensorineural and/or mixed)^11^, as well as in relation to the degree (mild, moderate, moderately severe, severe and profound)^12^.

Knowledge about the hearing changes and early diagnosis concerning hearing loss helps in the prognosis, reduces the damages caused by disease development, reduces sensorial deprivation, improves inclusion in society and, it also contributes to improving quality of life. Thus, it is relevant to investigate how such hearing loss happened and its relationship with the HIV infection/AIDS in affected people.

Therefore, we ask: how have the HIV-associated hearing changes been approached in the scientific literature? Which factors are associated with the hearing loss in patients with HIV/AIDS?

It is worth stressing that there is a need for a hearing health policy encompassing the particularities associated with prevention, early diagnosis and treatment of hearing disorders in the Brazilian public healthcare system.

In pursuing these answers, our study aimed at identifying the factors associated with hearing loss in people with HIV/AIDS in the literature.

## METHOD

This is an Integrative Literature Review, which enables the abstracting of previous studies and establishing conclusions based on the critical review of the studies outlines, enabling one to summarize the evidence available about the theme being investigated^13^.

The stages followed in order to operate this review involved: problem identification, creation of a research protocol, defining the information to be extracted from the papers selected, selection of papers, analysis, presentation and discussion of results^13^, ^14^.

The papers were searched in four virtual databases, namely: *ISI Web of Knowledge* (*Institute for Scientific Information*), PUBMED/MEDLINE (*Medical Literature Analysis*), LILACS (Literatura Latino-Americana em Ciências de Saúde - Health Sciences Latin American Literature) and SCIELO (*Scientific Electronic Library Online*), using the following keywords listed in the Health Sciences Keywords: *hearing loss, hearing disorders* and *deafness*, individually associated to the *HIV* keyword.

The inclusion criteria were: original papers, open access papers, papers published in Portuguese, Spanish, English and French, without time constraints. The exclusion criteria were: repetitive papers, reviews, meeting proceedings, opinion papers, editorials, theses, dissertations, epidemiological bulletins, papers which do not directly approach the theme of this study.

**Figure 1:**
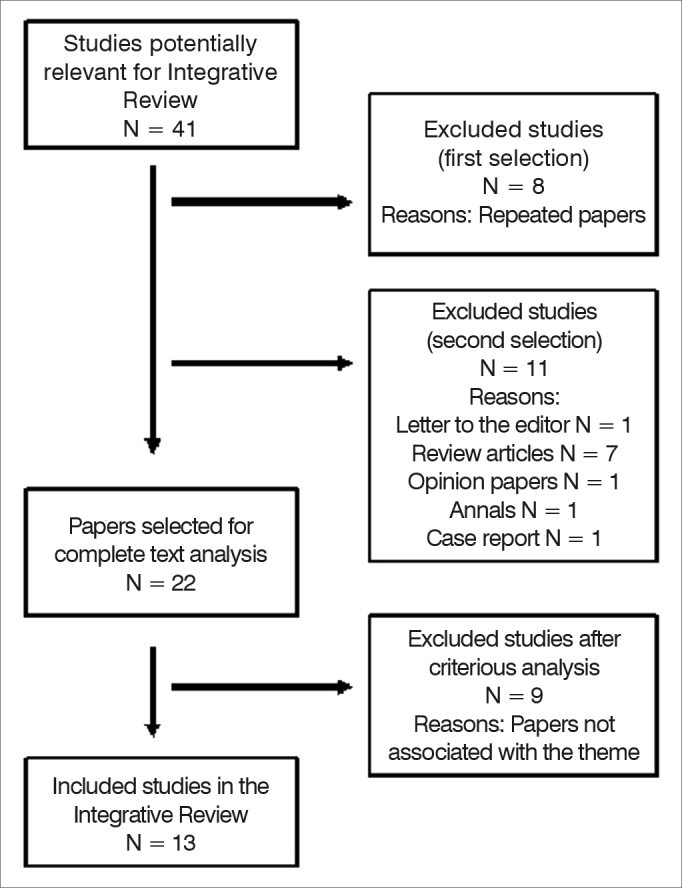
Summary of the process used to select the papers used in the integrative review of the literature.

We found a total of 41 publications. After reading the title and summary, we took off repeated papers and those which did not meet the inclusion criteria. There were 22 papers left for complete text analysis, and of these we took nine papers off because they were not associated with the theme being studied. The sample was made up of 13 scientific papers, as per shown by Figure 1 below.

In order to extract the data from the selected studies, we created a table with columns, bearing the following information: year of publication, authors, title, journal, country, type of study, study design, settings, target population, hearing loss-HIV association.

The data was grouped according to the similarities and differences, making up a finding which was interpreted and supported by other pertaining papers. All the studies selected are listed on Chart 1 and have references in this text.

**Chart 1:** Selected papers on hearing loss and associated factors in people with HIV/AIDS, according to author, year and country of publication, title, study type, study goals, and factors associated with hearing loss.

Author	Country/year	Title	Study type	Objective	Associated factors
Vieira ABC, Greco DB, Teófilo MMM, Gonçalves DU.^15^	Brazil/2008	Otoneurological manifestations associated with the antiretroviral therapy	Case-control study	Assess a possible correlation between ototoxicity and antiretroviral therapy	Ototoxicity Virus action
Schouten JT, Lockhart DW, Rees TS, Collier AC, Marra CM.^9^	USA/2006	A prospective study of hearing changes after beginning Zidovudine or Didanosine in HIV-1 treatment-naïve people	Prospective study	To assess the effects of Zidovudine and Didanosine causing hearing loss	Ototoxicity Virus action
Matas CG, Marcon BA, Silva SM, Gonçalves IC.^16^	Brazil/2010	Hearing assessment in the Acquired Immunodefciency syndrome	Case-control study	Characterize the audiological manifestations in HIV/AIDS adults and compare the results from individuals who were exposed to those who were not exposed to antiretroviral treatment	Ototoxicity Virus action
Palacios GC, Montalvo MS, Freire MI, Leon E, Alvarez MT, Solorzano F.^17^	México/2008	Audiological and vestibular findings in a sample of Human Immunodeficiency Virus type-1-infected Mexican children under Highly Active Antiretrovi-ral Therapy	Case-control study	Assess audiological and vestibular disorders in children with HIV who received highly active antiretroviral therapy	Opportunistic diseases Ototoxicity
Meynardi JL, Amrani ML, Meyohas MC, Fligny I, Gozlan J, Rozenbaum W, Roullet E, Frottier J.^18^	France/1997	Two cases of cytomegalovirus infection revealed by hearing loss in HIV-infected patients	Case study	To report two cases in which the cytomegalovirus infection was revealed by the involvement of the 8th cranial nerves	Opportunistic diseases
Colugnati FB, Staras SAS, Dollard SC, Cannon MJ.^19^	USA/2007	Incidence of cytomegalovirus infection among the general population and pregnant women in the United States	Cohort study	Obtain estimates of infection forces, basic reproduction rates, and the mean age of CMV infection	Opportunistic diseases
Chandrasejhar SS, Sujana S, Connelly PE, Brahrnbhatt SS, Shah CS, Kloser PC, Baredes S.^20^	USA/2000	Otological and Audiological Evaluation of Human Immunodeficiency Virus-Infected Patients	Case study	Quantify the incidence of ear disease in HIV-infected patients	Ototoxicity
Khanna N, Nüesch R, Buitrago-Tellez C, Battegay M, Hirsch HH.^21^	Switzerland/2006	Hearing Loss after Discontinuing Secondary Prophylaxis for Cryptococcal Meningitis: Relapse or Immune Reconstitution?	Case study	Report on the case of a 26-year-old HIV-infected men with right-side hearing loss two months after interruption of the secondary prophylaxis because of cryptococ-cosis and meningitis.	Opportunisti diseases and virus action
Molyneux EM, Tembo M, Kayira K, Bwanaisa L, Mweneychanya J, Njobvu A, Forsyth H, Rogerson SR, Walsh AL, Molyneux ME.^22^	Malawi/2003	The effect of HIV infection on pediatric bacterial meningitis in Blantyre, Malawi	Case-control study	Compare the presentation, progress and results from acute bacterial meningitis in HIV-positive and negative children	Opportunistic diseases and virus action
Klemm E, Wollina U.^23^	Germany/2004	Otosyphilis: report on six cases	Cross-sectional/longitudinal cohort study	Investigate the frequency, clinical presentation and results from otosyphilis	Opportunistic diseases and virus action
Soumare M, Seydi M, Ndour CT, Fall N, Dieng Y, Sow AI, Diop BM.^24^	Senegal/2005	Epidemiological, clinical, etiological features of neuromeningeal diseases at the Fann Hospital Infectious Diseases Clinic, Dakar	Retrospective study	Determine the prevalence of cerebromeningeal diseases, and describe epidemiological data, clinical and etiological characteristics	Opportunistic diseases
Shahab I, Osbone BM, Butler JJ.^25^	USA/1994	Nasopharyngeal Lymphoid Tissue Masses in Patients with Human Immunodeficiency Virus	Histology findings and clinical correlation	Check for a correlation between HIV-1 and nasopharyngeal lymphoma	Opportunistic diseases
Wenzel GI, Götz F, Lenarz T, Stöver T.^26^	Germany/2008	HIV-associated cerebral lymphocyte infltration mimicking vestibular schwannoma	Case study	Report the first case of HIV associated with primary cerebral lymphocytes.	Ototoxicity

## RESULTS

To analyze and discuss the occurrence of hearing disorders in HIV/AIDS patients and its associated factors in the selected papers, they were organized in topics. Since it was not possible to group the studies by methodological similarity, we chose to group the findings by adherence to theme, namely: “Virus action^9^, ^15^, ^16^, ^21^, ^22^ and ototoxicity”^9^, ^15^, ^16^, ^17^, ^20^, ^26^; and “Opportunistic diseases and hearing loss”^17^, ^18^, ^19^, ^21^, ^22^, ^24^, ^25^.

### Virus action and ototoxicity

In 1995, authors had already found a high incidence of audiological changes in HIV-positive patients^27^. Notwithstanding, they emphasize the difficulties in stating whether these findings would be due to the direct action of the virus on the hearing pathways or a consequence of the ototoxicity induced by the medication used by these patients.

In a recent study involving adult patients treated and not treated by ARV therapy, the audiological manifestations (hearing loss, tinnitus and dizziness) were more frequent than the vestibular changes in patients treated with ARV. There were thirty-nine treatment regimens utilized in the group of treated patients, and five of those treatment regimens were associated with otoneurological signs (Didanosine-Lamivudine-Lopinavir/r; Zidovudine-Lamivudine-Efavirenz; Zidovudine-Lamivudine-Nevirapine; Stavudine-Lamivu-dine-Lopinavir/r; Zidovudine-Didanosine-Nelfinavir), and there were no significant correlation between the ototoxicity and the ARV treatment^15^. Moreover, in 33 individuals using antiretroviral with peripheral T CD4+ cells below 200 cel./ml, we also did not correlate hearing loss to using AZT and ddI. Thus, the initial hearing involvement in adults may be associated with the direct action of the virus on the central auditory system^9^.

However, adult HIV-positive individuals between 18 and 58 years of age, exposed to ARV treatment, had changes suggestive of peripheral auditory pathway involvement and also had high frequency hearing thresholds compromised^16^ when compared to the untreated group.

HIV/AIDS patients may have changes in their conventional audiological assessment, high-frequency audiometry, otoacoustic emissions, otoacoustic emission suppression and in auditory evoked potentials, suggesting involvement in the periphery auditory pathway as well as in the central auditory pathway^7^, ^16^.

High-frequency audiometry assesses hearing in the range above 8,000 Hz - between 9,000 and 20,000 Hz - and does not have standardized results, such as in a conventional audiometry - between 250 and 8,000 Hz. Nevertheless, people with HIV/AIDS are the first to be affected with some ear disorders, showing an important element used in early diagnosis^28^.

High-frequency audiometry is very useful to follow up patients exposed to intense noise: those who use ototoxic medication; as well as those exposed to degenerative etiological agents; nonetheless, some factors prevent this test to be part of the clinical routine, they are: we still lack a normality standard to test these frequencies, specificity in the equipment calibration patterns and the methodology employed, which may impact the results obtained^29^.

We stress the study carried out with 23 children (ranging between 5 months and 16 years of age) diagnosed with HIV/AIDS, receiving highly active ARV therapy. Tonal audiometry was carried out in 12 children with more than 4 years of age, and 33% had hearing loss, two were of conduction type. The brainstem responses were measured in all 23 children, suggesting a conductive hearing loss in six and sensorineural in two. Concerning the brainstem auditory responses, they had changes at different levels of the auditory pathways^19^, showing that children with HIV and using ARV therapy are prone to having auditory changes, just as well as adults are.

Thus, there are peripheral auditory pathway changes affecting people with HIV/AIDS associated with ototoxicity and to the high viral load or virus action. One may suppose that such fact is due to the morphological arrangement of the nervous fibers, since they have characteristics of the hair cells and/or of neurons, in responding in a specialized way to certain frequencies, which is called tonotopy. In tonotopy, the nervous fibers coming from the cochlear apex and forming the central region of the cochlear nerve are responsible for the transmission of low frequencies, while those fibers coming from the base and make up the periphery region of the nerve, are responsible for transmitting high frequencies, showing that, in current times it is not possible to be clear about the agent causing hearing loss in these people^30^.

### Opportunistic diseases and hearing loss

Concerning opportunistic diseases associated with hearing loss, we have the prevalence of central nervous system infections by cytomegalovirus (CMV), which must be considered as a cause for hearing loss in HIV-infected patients^18^.

CMV is a broadly distributed infectious agent in the general population, belonging to the herpes virus family, and it is a frequent cause of infections in human beings. This virus has periods of activation and latency, and once the person is infected, the virus remains indefinitely in the host's body, and it can be reactivated at any time, especially in the presence of immunosuppression agents^31^.

CMV incidence in the United States population is 1.6 infections for every 100 persons per year - and these are individuals with low family income. There are about 27,000 new CMV infections among serum-negative pregnant women every year^19^. In Brazil, serum-prevalence studies in the adult population have shown approximately 90% of positivity in the city of São Paulo^32^, as well as in Santa Catarina state^33^.

Upon studying 50 cases of patients infected with HIV aiming at characterizing the occurrence of ear diseases, we used a questionnaire, ear exam, audiological assessment and reviewing hospital charts. Among the otological manifestations we had: ear fullness (34%), tinnitus (26%), hearing loss (29%), otalgia (23%) and otorrhea (5%). Otitis media was also a frequent finding in these patients, and the sensorineural hearing loss was more severe in HIV patients, but it was not associated with the routine medication used to treat HIV/AIDS. The authors concluded that auditory disease affects up to 33% of HIV-infected patients, and sensorineural hearing loss is more severe in this specific group^20^.

We know that otitis may cause temporary peripheral hearing loss, and it must be diagnosed as soon as possible in order to install proper medical treatment. According to the National Institute of Health of the USA, there are about 75% of adults with AIDS with some kind of hearing disorder arising from opportunistic infections, its treatment and ototoxic effect^34^.

There is a case report of a male patient infected with HIV who had hearing loss on the right side two months after secondary prophylaxis for cryptococcuc meningitis. The authors associate the unilateral hearing loss of adult patients with cryptococcus meningitis and the treatment of HIV-associated infections^21^.

HIV-infected children were assessed in order to measure their audiological skills and vestibular disorders, considering that they were under highly active antiretroviral treatment. We included 23 patients with mean age of 4.5 years; audiometry was carried out in 12 children with more than 4 years of age. Four children had sensorineural hearing loss and two of them had conduction-type hearing loss. Brainstem responses were measured in all the patients, suggesting conductive-type hearing loss in six children and sensorineural hearing loss in two. The ones diagnosed with conductive hearing loss had a past of acute or chronic otitis media. These results suggest that children with HIV-1 must be audiologically assessed as soon as possible in order to reduce its impact on their psychosocial development^17^. We have also noticed that HIV+ children develop bacterial meningitis, have a high mortality rate, and are more prone to recurrent diseases^22^.

One epidemiological study carried out in order to study the frequency and clinical presentation of otosyphilis results in developed countries confirmed that it still is a complication of syphilis in these countries. Thus, we can state that this disease is a known cause of sensorineural hearing loss and has a higher prevalence among HIV patients. The otological involvement by *Treponema pallidum* happens in the tertiary syphilis. Initial symptoms may include uni or bilateral sensorineural hearing loss, usually of rapid progression and, often times sudden. Tinnitus, ear pressure and labyrinthine symptoms may also be present. The audiometric curve usually shows a drop in the low frequencies, suggesting endolymphatic hydrops^3^.

Cerebromeningeal diseases may be associated with hearing loss. In a retrospective study carried out in Senegal, with the aim of establishing the prevalence of cerebromeningeal involvement in hospitalized patients and describe epidemiology, clinical signs and symptoms and disease etiology, there were 470 cases identified - 89 children and adults had HIV and numerous opportunistic disorders, such as meningeal syndrome, coma, seizure, focal neurological failure, cranial nerve dysfunction, cerebral malaria, purulent meningitis, cryptococcal meningitis, tuberculous meningitis, intracranial abscess, toxoplasmosis, cerebromeningeal hemorrhage. Of the 89 investigated, 22 survived and among these there were five with hearing loss. The study concluded that the labs must have greater technical capacity to diagnose the opportunistic infections presented by these patients, as well as involve the many experts to manage cerebromeningeal sequelae^24^.

There are also two studies which did not reach conclusions as to the causes of the hearing loss. In the first one, with nine HIV-positive men, between 25 and 42 years of age, there were five with hearing loss^25^. The second is a case study with a 36-year-old man with unilateral progressive hearing loss, high ipsilateral tinnitus, sudden hearing loss followed by ipsilateral paralysis. In an MRI they suspected of a vestibular schwannoma; however, when they ordered the anti-HIV they found the patient was serum-positive. The authors recommended a thorough exam to investigate the cause of the hearing loss^26^.

Knowing that, every day, HIV/AIDS kills 6,000 people and infects some 8,200^35^ more and infection by opportunistic agents is a constant concern of patients with HIV, we stress the need to pay special attention to the early diagnosis and prevention of hearing loss disorders.

## CONCLUSIONS

We conclude that there is still no consensus in the studies we analyzed, concerning a determining factor for hearing disorders and hearing loss in persons who live with HIV/AIDS, and these changes are related to multiple factors. Among them we stress a high viral load, direct virus action on the cochleovestibular system, the use of TARV and opportunistic infections - having cytomegalovirus, meningitis, otosyphilis and treatments, the ones most correlated to the hearing disorder and ototoxicity.

Therefore, it was not possible to find a direct association between the ARV treatment and the hearing loss. Notwithstanding, we found hearing disorders in the population investigated, and their associations and causes are still not clear enough and concluding, which fosters the need for studies investigating the hearing loss in this population in a more in-depth manner.

To those adult and children with HIV/AIDS, we recommend hearing monitoring in Hearing Reference Centers, minimizing the effects from hearing-loss-causing factors, thus reducing the psychosocial impact and providing a better quality of life to these people.

Therefore, we require new studies, going more in-depth concerning the hearing disorders of patients with HIV/AIDS, which may become references for the initiatives of training and bringing awareness to healthcare professionals as to its problems and control.
